# Assessment and management of obesity and metabolic syndrome in children with CKD stages 2–5 on dialysis and after kidney transplantation—clinical practice recommendations from the Pediatric Renal Nutrition Taskforce

**DOI:** 10.1007/s00467-021-05148-y

**Published:** 2021-08-10

**Authors:** Stella Stabouli, Nonnie Polderman, Christina L. Nelms, Fabio Paglialonga, Michiel J. S. Oosterveld, Larry A. Greenbaum, Bradley A. Warady, Caroline Anderson, Dieter Haffner, An Desloovere, Leila Qizalbash, José Renken-Terhaerdt, Jetta Tuokkola, Johan Vande Walle, Vanessa Shaw, Mark Mitsnefes, Rukshana Shroff

**Affiliations:** 1 1st Department of Pediatrics, Hippokration Hospital, Aristotle University Thessaloniki, 49 Konstantinoupoleos Str, 54642 Thessaloniki, Greece; 2 British Columbia Children’s Hospital, Vancouver, Canada; 3 University of Nebraska, Kearney, NE USA; 4 Fondazione IRCCS Ca’ Granda Ospedale Maggiore Policlinico, Milan, Italy; 5 Emma Children’s Hospital, Amsterdam University Medical Center, Amsterdam, The Netherlands; 6 Emory University, Atlanta, GA USA; 7 Children’s Healthcare of Atlanta, Atlanta, GA USA; 8 Children’s Mercy Kansas City, Kansas City, MO USA; 9 University Hospital Southampton NHS Foundation Trust, Southampton, UK; 10 Children’s Hospital, Hannover Medical School, Hannover, Germany; 11 University Hospital Ghent, Ghent, Belgium; 12 Great Northern Children’s Hospital, Newcastle upon Tyne, UK; 13 Wilhelmina Children’s Hospital, University Medical Center Utrecht, Utrecht, The Netherlands; 14 Children’s Hospital and Clinical Nutrition Unit, Internal Medicine and Rehabilitation, University of Helsinki and Helsinki University Hospital, Helsinki, Finland; 15 University Hospital Ghent, Ghent, Belgium; 16 University College London Great Ormond Street Hospital Institute of Child Health, London, UK; 17 Division of Nephrology and Hypertension, Cincinnati Children’s Hospital Medical Center, Cincinnati, OH USA

**Keywords:** Obesity, Metabolic syndrome, Chronic kidney disease, Kidney transplantation, Diet, Physical activity, Screen time, Sleep, Clinical practice recommendations, Pediatric Renal Nutrition Taskforce

## Abstract

Obesity and metabolic syndrome (O&MS) due to the worldwide obesity epidemic affects children at all stages of chronic kidney disease (CKD) including dialysis and after kidney transplantation. The presence of O&MS in the pediatric CKD population may augment the already increased cardiovascular risk and contribute to the loss of kidney function. The Pediatric Renal Nutrition Taskforce (PRNT) is an international team of pediatric renal dietitians and pediatric nephrologists who develop clinical practice recommendations (CPRs) for the nutritional management of children with kidney diseases. We present CPRs for the assessment and management of O&MS in children with CKD stages 2–5, on dialysis and after kidney transplantation. We address the risk factors and diagnostic criteria for O&MS and discuss their management focusing on non-pharmacological treatment management, including diet, physical activity, and behavior modification in the context of age and CKD stage. The statements have been graded using the American Academy of Pediatrics grading matrix. Statements with a low grade or those that are opinion-based must be carefully considered and adapted to individual patient needs based on the clinical judgment of the treating physician and dietitian. Research recommendations are provided. The CPRs will be periodically audited and updated by the PRNT.

**Supplementary Information:**

The online version contains supplementary material available at 10.1007/s00467-021-05148-y.

## Introduction

Optimizing cardiovascular (CV) health is one of the major treatment goals in patients with chronic kidney disease (CKD) since CV disease contributes to significant morbidity and mortality [1]. Unfortunately, children with CKD have an increased prevalence of traditional CV risk factors [2], even in the absence of overt kidney dysfunction. The cluster of cardio-metabolic risk factors, referred to as metabolic syndrome (MS), affects children at all stages of CKD, including those on dialysis and after a kidney transplant, due to the worldwide obesity epidemic [3, 4].

The prevalence of MS in children with CKD is currently reported at 15–30% [2, 5, 6], higher than the 3.8–9.8% prevalence in European and United States (US) general adolescent populations [7, 8]. Registry data suggests that obesity may affect CKD progression, patient mortality, access to transplantation, and graft function [9–11]. Studies on MS in children with CKD and kidney transplantation highlight the adverse effects of cardio-metabolic risk factors, in addition to obesity, on outcomes [5, 6, 12–15]. Finally, emerging evidence that obesity can impair kidney function, even in otherwise healthy obese adolescents [16], highlights the importance of managing cardio-metabolic risk factors in children with pre-existing kidney diseases.

The Pediatric Renal Nutrition Taskforce (PRNT), an international team of pediatric nephrologists and pediatric renal dietitians, provides clinical practice recommendations (CPRs) on various aspects of the dietary management of children with CKD. In this CPR, we discuss the management of obesity and metabolic syndrome (O&MS) in children and adolescents with CKD stages 2–5 and on dialysis, as well as after kidney transplantation, focusing on non-pharmacological treatment (diet, physical activity and behavior modification). As with all CPRs produced by the PRNT, resources for the practical management of O&MS will be developed by the Taskforce during the dissemination phase of the guideline [17].

## Methods

The composition of the PRNT and the full development process for the CPRs and their purpose, search criteria, grading of evidence, and plans for audit and revision of the CPRs is described in previous PRNT guidelines [17]. PICO questions and literature search are described in the supplementary material. Consensus from consideration of the PICO questions led to specific recommendations.

### Clinical practice recommendations

1. How is O&MS defined?
  Children aged 2–5 years:
  We define overweight as weight-for-height for age > +2SD, using the World Health Organization (WHO) child growth standard chart.
  We define obesity as weight-for-height for age > +3SD, using the WHO child growth standard chart.
  Children aged > 5 years:
  We define overweight as body mass index (BMI) for age > +1SD, equivalent to BMI > 25 kg/m^2^ at 19 years, using the WHO growth reference chart or a country-specific growth chart.
  We define obesity as BMI for age > +2SD, equivalent to BMI > 30 kg/m^2^ at 19 years, using the WHO growth reference chart or a country-specific growth chart.
  Children aged 2–18 years:
  We define metabolic syndrome as the presence of overweight or obesity and at least 2 of 4 additional CV risk factors:
  - Systolic and/or diastolic office blood pressure (BP) ≥ 90th centile for age, sex and height or ≥ 130/80 mmHg, whichever is lower, or on anti-hypertensive medication
  - Fasting triglycerides ≥ 100 mg/dL (1.1 mmol/L) if age < 10 years, or ≥ 130 mg/dL (1.5 mmol/L) if age ≥ 10 years
  - Fasting high-density lipoprotein (HDL) < 40 mg/dL (1.03 mmol/L)
  - Fasting serum glucose ≥ 100 mg/dL (5.6 mmol/L) or known type 2 diabetes mellitus (T2DM)

1.1. We recommend using BMI-height-age to define overweight or obesity in children who are below the 3rd centile for height and have not reached their final adult height (Level B; moderate recommendation).

### Evidence and rationale

#### Definitions of MS in the general pediatric population

Several scientific organizations have provided consensus definitions for MS in children and adolescents [18, 19] (Table 1). Obesity, especially abdominal, is considered the main pathophysiological drive for the evolution of cardio-metabolic risk factors in healthy children and adolescents [18]. However, not all definitions of O&MS have included overweight or obesity as a prerequisite for the definition of metabolic syndrome. Further discrepancies among definitions are due to different thresholds for other components. The use of various definitions may result in different prevalence numbers and different attributable risk levels for future adverse cardio-metabolic outcomes [7].

**Table 1:** Definitions of metabolic syndrome used in studies in children with CKD

	Weiss et al. [20]	Cruz et al. [21]	International Diabetes Federation [18]	Expert panel on integrated guidelines for cardiovascular health and risk reduction in children and adolescents [19]	Pediatric Renal Nutrition Taskforce 2021 In children with CKD2-5, on dialysis and after transplantation
Definition	Any ≥ 3 of 5 criteria	Any ≥ 3 of 5 criteria	WC ≥ 90th plus any 2 ≥ criteria	Any ≥ 3 of 5 criteria	Overweight or obesity plus any 2 ≥ criteria
Overweight/obesity	BMI > 97th centile for age and sex (or BMI z score ≥ 2)	WC ≥ 90th centile for age, sex, and Hispanic ethnicity from NHANES III	6- < 10 yrs: WC ≥ 90th centile 10- < 16 yrs: WC ≥ 90th centile or adult cut off if lower > 16 yrs: WC ≥ 94 cm in Europid males or ≥ 80 cm in females or other ethnic specific values	BMI ≥ 85th centile or WC ≥ 90th centile	2-5 yrs: BMI > +2SD on growth charts > 5 yrs: BMI > +1SD on growth charts
High TG	> 95th age and sex centile	≥ 90th age and sex centile	10–< 16 yrs: ≥ 150 mg/dL > 16 yrs: ≥ 150 mg/dL or treatment for high TG	≥ 75 mg/dL if 0–9 years ≥ 90 mg/dL if ≥ 10 years	< 10 yrs: TG ≥ 100 mg/dL ≥ 10 yrs: ≥ 130 mg/dL
Low HDL	< 5th age and sex centile	≤ 10th age and sex centile	10–< 16 yrs: < 40 mg/dL > 16 yrs: < 40 mg/dL in males < 50 mg/dL in females or treatment for low HDL	≤ 40 mg/dL	≤ 40 mg/dL
High BP	> 95th age, sex and height centile	≥ 95th age, sex and height centile	10–< 16 yrs: ≥ 130/85 mmHg > 16 yrs: ≥ 130/85 mmHg or diagnosis of HTN	≥ 90th age, sex and height centile	≥ 90th age, sex, and height centile or ≥130/80 mmHg or on antihypertensive medication
Glucose intolerance	Glucose ≥ 140 mg/dL at 2 h OGTT	Glucose > 140 mg/dL (3 h OGTT)	FG ≥ 100 mg/dL or known T2DM	FG ≥ 100 mg/dL	FG ≥ 100 mg/dL

*Abbreviations*: *WC* waist circumference, *BMI* body mass index, *TG* triglycerides, *HDL* high-density lipoprotein, *BP* blood pressure, *HTN* hypertension, *OGTT* oral glucose tolerance test, *FG* fasting glucose, *T2DM* type 2 diabetes mellitus

The significance of a clustering of CV risk is highlighted by the hallmark Bogalusa Heart Study showing the effect of increasing number of childhood CV risk factors on atherosclerotic lesions [22]. Similarly, the Young Finns Study has shown that the number of CV risk factors in 12- to 18-year-olds was directly related to carotid intima media thickness (cIMT) measured in young adults at ages 33–39 years [23]. In the Pathobiological Determinants of Atherosclerosis in Youth Study, CV risk factors were associated with atherosclerotic lesions in the left anterior descending coronary artery, right coronary artery, and abdominal aorta in persons aged 15 to 34 years who died from external causes [24]. An AAP publication pointed out limitations of using MS definitions in pediatrics, including the instability of definitions over time and at transition from adolescence to adulthood [25].

#### Performance of previous O&MS syndrome definitions in CKD patients

O&MS is of particular concern in kidney transplant recipients (Table 2). Studies in adults have mostly used the National Cholesterol Education Program (NCEP) Adult Treatment Panel III (ATP III) definition for MS, but there is heterogeneity among the definitions used in pediatric studies (Table 1) [5, 6, 12–14, 20, 21, 27–29]. Nevertheless, most studies have found an association of O&MS with declining kidney function and CV outcome, independent of the definition used [5, 6, 12–14, 27]. No clear conclusion on the superiority of any single definition can be drawn as study designs have not provided head-to-head comparisons.

**Table 2:** Studies on CV and renal outcomes of MS in children with CKD and after kidney transplantation

Study/country/design	Population	MS definition	Risk factors for MS	MS prevalence	MS Outcome
Lalan et al. [5] USA Multi-center, observational prospective cohort study	472 children, median age ~12 years from the CKiD study (eGFR 30–90 mL/min/1.73 m^2^)	Overweight plus ≥ 2 criteria Expert Panel 2011	NR	15.1% (40% among OW, 60% among OB)	2 times greater OR of decline in eGFR >10% per year
Sgambat et al. [26] USA Single-center prospective, longitudinal cohort study	42 kidney transplant recipients from single center aged 3-20 years (mean eGFR at end of study 90.3 ± 3.1 mL/min/1.73 m^2^) 24 healthy controls	Abdominal obesity plus ≥ 2 criteria Modified Expert Panel 2011 ATP-III if > 19 yrs	The prevalence of obesity as detected by WHr was significantly higher (2.3–5.1 times) than by WC alone	33.3%, 29.7%, 30.3% at 1, 18, and 30 months post-transplant	- BMI-obesity, WHr-obesity and MS had 3.7 ± 1.9, 2.8 ± 1.3, and 3.6 ± 1.8 times higher ORs for post-transplant LVH. WC-obesity was not a predictor. - BMI-obesity, WHr-obesity, WC-obesity, and MS had 1.5 ± 0.39, 1.4 ± 0.49, 1.6 ± 0.51, and 1.3 ± 0.58 times higher ORs for post-transplant worse myocardial longitudinal strain (%). - In the models, there were also higher ORs for high BP for LVH and high BP and eGFR for strain.
Sgambat et al. [27] USA Single-center prospective, longitudinal cohort study	42 kidney transplant recipients from single center aged 3–20 years (mean eGFR at end of study 90.3 ± 3.1 mL/min/1.73 m^2^) 24 healthy controls	Abdominal obesity plus ≥ 2 criteria Modified Expert Panel 2011 ATP-III if > 19 yrs	NR	33.3%, 29.7%, 30.3% at 1, 18, and 30 months post-transplant	Among the 21 AA transplant patients, MS was independently associated with a 0.03 ± 0.01-mm increase in cIMT.
Tainio et al. [12] Finland Single-center retrospective cohort study	210 kidney transplant recipients from single center median age 4.5 years (range 0.7–18.2)	Any of ≥ 3 criteria Modified for the study AHA 200	NR	19% at 1.5 years and 14.2 at 5 years post-transplant	Higher ^51^Cr-EDTA GFR decline in MS at 1.5 years (ml/min/1.73 m^2^), but no difference at 5 years post-transplant
Wilson et al. [2] USA Multi-center, observational prospective cohort study	586 children 1–16 years from the CKiD study (eGFR 30–90 mL/min/1.73 m^2^)	≥ 3 CV risk factors	NR	13%	Nephrotic-range proteinuria was associated with 2.04 higher odds of having more CV risk factors.
Wilson et al. [13] USA Retrospective multi-center study	234 kidney transplant recipients from 6 centers in the Midwest Pediatric Nephrology Consortium aged 12.1 ± 5.16 years (mean eGFR at end of study 87.3 ± 28.3 mL/min/1.73 m^2^)	Any of ≥ 3 criteria Weiss 2004 [20]	Factors associated with incident metabolic syndrome included pretransplant BMI > 85th centile and cyclosporine	18.8% at time of transplant 37.6% at 1-year post-transplant (40% among overweight and 74.5% among obese)	- 2.6 times higher OR for post-transplant LVH - 3 times higher OR of eccentric LVH hypertrophy post-transplant - 55% in MS vs. 32% in those without - Mean LVMI was 48.3 g/m^2.7^ in MS vs. 40.0 g/m^2.7^ (p = 0.0008) without MS
Maduram et al. [14] USA Retrospective single-center cohort study	58 kidney transplant recipients from single center aged 11.2 ± 5.1 years	Any of ≥ 3 criteria Age-modified ATP-III	Prevalence significantly higher 68% in steroid group vs. 15% in steroid withdrawal group	38%	Lower GFR in children at 1-year post-transplant (65) vs those without MS (65 ± 19 vs. 88 ±25 mL/min/1.73 m^2^) in both steroid and steroid withdrawal groups

*Abbreviations*: *GFR* glomerular filtration rate, *OW* overweight, *WHr*, waist-to-height ratio, *WC* waist circumference, *BMI* body mass index, *BP* blood pressure, *LVH* left ventricular hypertrophy, *cIMT* carotid intima media thickness, *CV* cardiovascular, *LVMI* left ventricular mass index

#### Definition of O&MS components by the PRNT

**Fig. 1:**
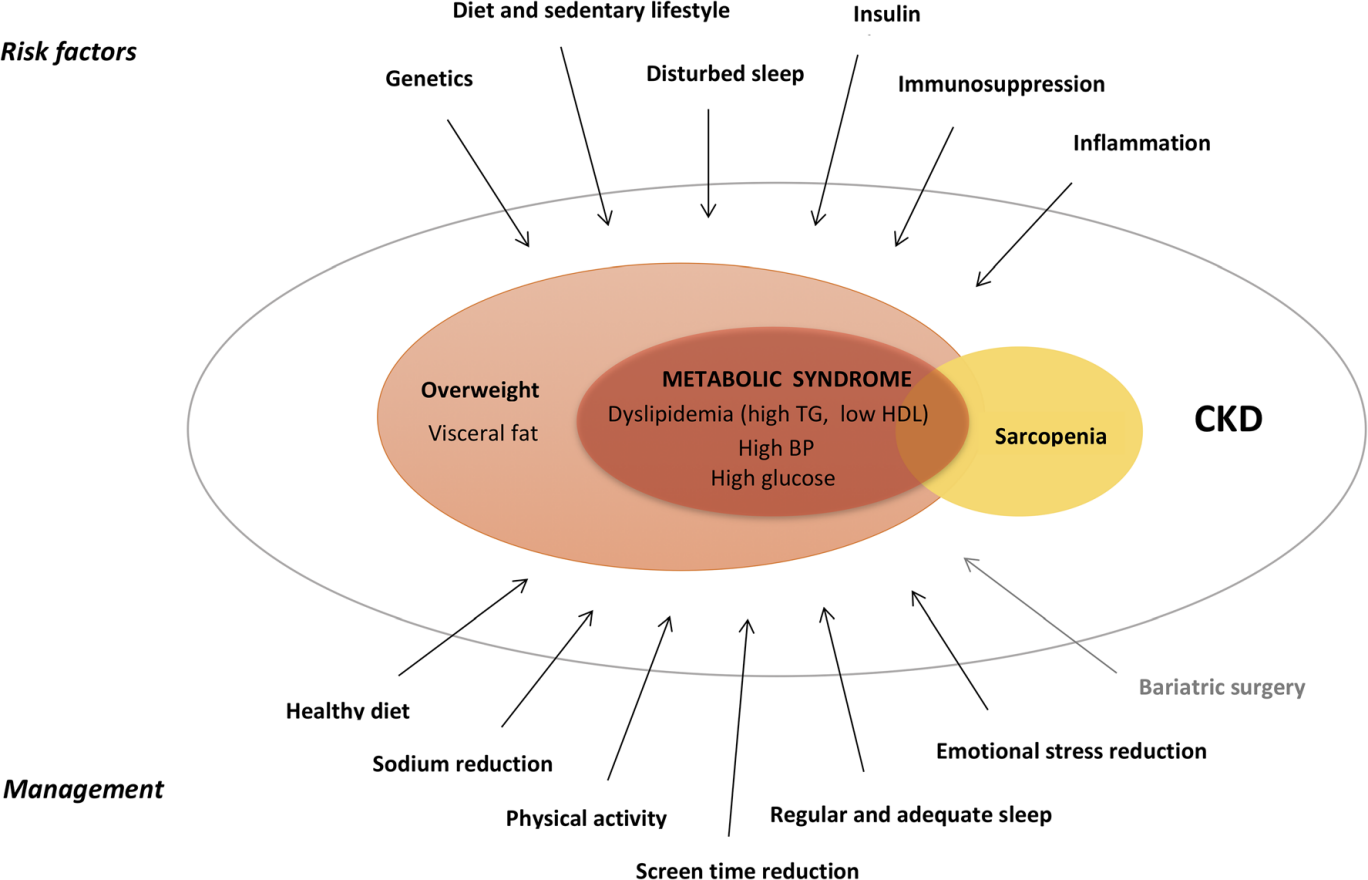
Traditional and disease-related risk factors and management of O&MS in CKD patients. First line (black) and second line (gray) treatment

Children with CKD have an increased prevalence of cardio-metabolic risk factors, independent of weight status [26]. Therefore, using overweight or obesity as a necessary criterion to define MS in children with CKD is important in order to emphasize the additive effect of adiposity on the cardio-metabolic risk profile, although it is not possible to differentiate the discrete impact of kidney disease and obesity in a given individual (Fig. 1). The definition proposed by the PRNT is consistent with the concept proposed by the International Diabetes Federation and AAP that obesity and overweight are central to the definition of MS and CV risk clustering [18, 25]. In this PRNT CPR, we use BMI for age or BMI-height-age to define overweight and obesity. Although central adiposity is well recognized for identifying MS, only a few studies in pediatric CKD patients have used waist circumference or waist-to-height ratio to define obesity with conflicting results on the clinical superiority of these measurements over BMI; these measurements need further research before being widely recommended in clinical practice [26, 30]. In addition, waist measurement in children on peritoneal dialysis (PD), or in those with an increased abdominal girth, such as patients with autosomal recessive polycystic kidney disease, nephrotic syndrome and ascites, and those on steroid treatment, may not represent true visceral adiposity, thereby limiting the routine use of waist circumference and waist-to-height ratio in all children with CKD. The PRNT Assessment of Nutritional Status CPR previously discussed the rationale of using BMI-height-age for children shorter than the 3rd height centile who had not reached their final height [31]. Finally, data from the Pediatric Growth and Development Special Study (PGDSS) from the US Renal Data System showed increased mortality rates and decreased access to transplantation in obese children using BMI SDS values, supporting the clinical value of BMI to define obesity [10, 11].

Children with CKD are at increased risk for CV morbidity and mortality [1]. Assuming that the proposed thresholds defining MS components would also serve as thresholds for treatment, it was the consensus among PRNT members to introduce lower thresholds for these parameters in children with CKD2-5D and after transplantation than in healthy children for several MS components.

Height BP centile thresholds for O&MS should more accurately reflect the BP thresholds for children and adolescents with CKD and compromised growth. The 2021 Kidney Disease Improving Global Outcomes (KDIGO) clinical practice guideline on the management of BP in CKD introduced a BP target < 130/80 mmHg in kidney recipients or lower if tolerated in non-transplant adult CKD patients [32]. In children, evidence is limited. The European Society of Hypertension guidelines recommend BP targets lower than the 50th and 75th centiles for age, sex, and height in proteinuric and non-proteinuric pediatric CKD patients, respectively [33, 34]. These recommendations are based on the ESCAPE study, a large, randomized trial on intensified BP control in children with CKD showing slower progression of CKD when using these BP targets [35]. Also, in the few available studies in children with CKD that examined the effect of MS on subclinical target organ damage or disease progression, the use of 95th BP centile for age, sex, and height was consistently associated with adverse associations. Thus, lower BP limits might be considered in this population vulnerable to adverse CV outcomes. The use of the 90th centile threshold in children, when it is lower than 130/80 mmHg (the therapeutic BP target for adult CKD patients according to KDIGO), has been considered appropriate to be included in the current definition of MS in children with CKD2-5D and after transplantation.

For triglycerides and HDL cholesterol, the thresholds for healthy children are used for the definition of MS [19]. This is despite the fact that HDL molecules are dysfunctional in CKD patients and have reduced protective or even a pro-atherogenic vascular effect [36]. With regard to glucose metabolism, it seems prudent to maintain the diagnostic criteria for diabetes [37] in children with CKD and O&MS.

2. How is O&MS assessed?
  2.1. Calculate BMI or weight-for-height and plot on centile growth charts (level A; strong recommendation)
    2.1.1. Calculate z-scores (standard deviation scores (SDS)) to complement growth chart plots (level X; strong recommendation).
    2.1.2. Utilize trends in growth parameters to assist clinical decision-making (level D; weak recommendation).
  2.2. Measure BP, fasting TG, HDL, and glucose levels in children with CKD2-5D and after transplantation if BMI > +1 SD (level A; strong recommendation).
  2.3. Evaluate for MS risk factors, including focused history and physical exam, biochemical measurements for comorbidities, and assessment of cardio-metabolic risk factors (level C; weak recommendation).
  2.4. Evaluate lifestyle habits, including diet, physical activity, sleep, and screen time (level C; weak recommendation).
  2.5. The frequency of assessment should be individualized based on the child’s CV risk factors, disease severity and progression and the presence of comorbidities (Ungraded).

### Evidence and rationale

#### O&MS assessment

The assessment of children with CKD and O&MS is described in Table 3. A detailed document on the assessment of nutritional status in children with CKD has been published by the PRNT [31]. These CPRs suggest the minimum frequency of assessment given that children with CKD and O&MS are at higher risk for CV disease. Regular assessment of BP status by Ambulatory Blood Pressure Monitoring (ABPM), in addition to office BP measurement, has already been incorporated into the routine care of CKD patients [32, 33, 38].

**Table 3:** Assessment of patients with CKD stages 2–5 on dialysis and after kidney transplantation with O&MS

Assess	Suggested minimum Interval	
	CKD2-5 and kidney transplants	CKD5D
Anthropometry		
Euvolemic weight, height, length, weight-for-height, BMI for age, BMI-height-age, SDS	1–3 months	Monthly
BMI trends plotted on centile growth charts	1–3 months	Monthly
Medical history		
Family history of obesity, diabetes, hypertension, hyperlipidemia, cardiovascular disease	Yearly	Yearly
Perinatal history, primary disease, age of disease onset	At initial visit	At initial visit
Snoring and sleep apnea history, sleep duration, history of NAFLD, PCOS, mental disorders, concurrent disease (endocrine, cardiac, neurological, systemic, e.g., lupus), symptoms or history of target organ damage, past and current treatments, compliance and side effects	6–12 months	6–12 months
Physical exam		
Assessment may include for cushingoid features, skin for acanthosis nigricans, acne, hirsutism, eye examination for cataract or pseudotumor cerebri, ankle, foot, knee pain, joint dysfunction,	6–12 months	6–12 months
neurodevelopmental assessment, features of syndromic obesity (e.g., Bardet-Biedl syndrome)	At initial visit	At initial visit
Cardio-metabolic risk factors		
BP:		
Office BP measurement	At each visit	At each visit
ABPM	6–12 months	6–12 months
Lipids:		
Triglycerides	3–4 months	3–4 months
HDL		
Glucose metabolism:		
Fasting glucose	3–4 months	3–4 months
HbA1C	If glucose > 100 mg/dL, reassess every 6–12 months	If glucose > 100 mg/dL, reassess every 6–12 months
Additional risk factors:		
Transaminases	6–12 months	6–12 months
Uric acid	3–4 months	3–4 months
Dietary assessment		
Food record or food recalls	3–6 months	3–4 months
Dietetic contact	At each visit	At each visit
Physical activity		
Record frequency, duration, intensity of PA	3–4 months	1–3 months
Lifestyle habits		
Daily screen time, leisure activities	3–4 months	3–4 months
Echocardiography		
Evaluation for LVH	Yearly	Yearly

*Abbreviations*: *BMI* body mass index, *SDS* standard deviation score, *NAFLD* nonalcoholic fatty liver disease, *PCOS* polycystic ovary syndrome, *BP* blood pressure, *ABPM* ambulatory BP monitoring, *HDL* high-density lipoprotein, *HgA1C* hemoglobin A1c, *PA* physical activity, *LVH* left ventricular hypertrophy

#### Risk factors and comorbidities

Genetic, environmental, and perinatal factors determine the risk of O&MS in healthy children. Extensive evidence has highlighted the role of modifiable risk factors for O&MS, including poor nutritional habits, physical inactivity, excessive screen time, and short sleep duration, as well as non-modifiable risk factors, including parental obesity, maternal gestational diabetes, low birth weight, and rapid catch-up growth [25]. Obesity and insulin resistance share common pathogenetic mechanisms for causing comorbidities including non-alcoholic fatty liver disease (NAFLD), polycystic ovary syndrome (PCOS), obstructive sleep apnea, hyperuricemia, anxiety, and depression [25, 39]. In a large cohort of obese children, high levels of uric acid were independently associated with lower glomerular filtration rate (GFR) and NAFLD [40]. Although hard CV outcomes related to O&MS may not be seen until adulthood, subclinical organ damage may start from childhood. Left ventricular hypertrophy (LVH) is common and its prevalence increases with the number of CV clustering factors in addition to obesity [41].

Risk factors for the development of O&MS in children with CKD are those reported in the general pediatric population, including poor nutritional habits, lack of physical activity, and excessive screen time. Poor nutritional habits, including high energy intake and fast-food consumption, were reported in children with non-dialysis CKD in the Chronic Kidney Disease in Childhood (CKiD) cohort and are comparable to the general pediatric population [42, 43]. Moreover, children in the CKiD cohort had more sedentary behaviors compared to healthy youth from the National Health and Nutrition Examination Survey (NHANES) [44]. Hyperuricemia is frequent in children with CKD and associated with increased BMI, hypertension, and albuminuria [45]. Perinatal factors, including birth weight and maternal diabetes and obesity that are known risk factors for O&MS in the general pediatric population, are also associated with a higher risk of developing congenital anomalies of the kidney and urinary tract (CAKUT) [46]. Thus, common perinatal factors increase the risk of CAKUT at birth and subsequently increase the risk of O&MS.

In children with CKD, additional risk factors for the development of O&MS include disease-specific factors, CKD-related dietary modifications, enteral tube feeding, and treatment-related effects (Fig. 1). Steroid use after kidney transplantation is a major risk factor for the development of O&MS [14]. In a multicenter, randomized, open-label study in pediatric kidney transplant recipients, steroid withdrawal was associated with a significantly reduced incidence of MS [29].

Treatment with mTOR inhibitors may promote severe dyslipidemia in children after kidney transplantation [47]. Moreover, children with specific underlying kidney diseases, including cystic kidney diseases, nephropathic cystinosis, and Bardet–Biedl and Alstrom syndromes, are at greater risk of developing new onset diabetes after transplantation as well as other cardio-metabolic risk factors and subsequently O&MS [47].

#### Obese sarcopenia

Despite dietary excesses, some obese children may have inadequate protein intake that may contribute to sarcopenia. Sarcopenia, a condition characterized by progressive and generalized loss of muscle mass and strength [48], is more commonly seen in the frail and elderly, but may also be present in young obese people with or without CKD [49]. Several risk factors can lead to muscle loss in CKD, including the kidney disease itself, the dialysis procedure, chronic low-grade inflammation, metabolic acidosis, and vitamin D deficiency that together increase protein catabolism, decrease protein synthesis, and lead to a negative protein balance [50]. In obese CKD patients, obesity per se may act as a pro-inflammatory factor due to adipocyte dysfunction, characterized by increased synthesis of cytokines and chemokines (adipokines) that occurs as a consequence of adipocyte hypertrophy and hypoxia [51]. In addition, metabolic acidosis acts as a potent stimulator of protein catabolism and can promote insulin, triggering a vicious cycle of worsening sarcopenia [51–53]. Obesity can mask underlying muscle wasting and, although uncommon in children, must be considered in the evaluation of CKD patients. Hand grip strength has been used to assess children at risk of obese sarcopenia [54].

#### Relations of O&MS with outcomes in children with CKD

Few studies in children with CKD and O&MS, largely in children after kidney transplantation, have studied the effect on outcomes such as graft function and subclinical CV damage (Table 2). In 234 kidney transplant recipients from 6 centers in the Midwest Pediatric Nephrology Consortium, MS associated with 2.6 times higher odds for post-transplant LVH with 3 times higher odds of eccentric LVH post-transplant [13]. The latter finding is consistent with a large single-center prospective study in adults showing association of MS with higher frequency of CV events in about 2,000 kidney recipients [55]. In non-dialysis CKD children from the CKiD cohort, the odds of LVH were 1.5-fold higher in boys and 3.1-fold higher in girls for each 1-unit higher BMI z-score [56]. In view of this evidence, along with data from the healthy pediatric population indicating the early effects of O&MS, we recommend that children with O&MS and CKD2-5D or after kidney transplantation should have yearly echocardiography (Table 3).

Increased cIMT has been associated with hypertension and dyslipidemia [57], and higher pulse wave velocity (PWV) with BMI and fat mass in pre-dialysis children [58], but the clinical value of these findings needs to be validated by future studies. Measurement of cIMT or PWV for assessment of vascular subclinical damage is not advised.

3. How is O&MS managed?
  3.1. We suggest a comprehensive multicomponent intervention that includes a nutrition care plan, physical activity prescription, and behavioral modification to reduce BMI and improve components of the MS (ungraded).
  3.2. Diet
    3.2.1. We recommend an individualized energy intake, adjusted for age, CKD stage, dialysis, and comorbidities, to achieve weight loss or weight maintenance in children without compromising their nutrition (ungraded).
    3.2.2. The nutrition care plan should aim to improve the overall diet quality, with an emphasis on an intake composed primarily of fruits and vegetables, whole grains, low- or non-fat dairy products, pulses (peas, beans, lentils), fish and lean meat, and avoidance of sugar-sweetened beverages, highly processed foods, and foods high in saturated fat (level B; weak recommendation).
    3.2.3. In children who are enterally tube fed, the energy content of the formula must be frequently reviewed and adjusted to avoid development of underweight or overweight (level B; moderate recommendation).
  3.3. Physical Activity
    3.3.1. We recommend that children engage in daily physical activity with intensity and duration individualized according to age, physical tolerance, CKD stage, and comorbidities (level B; moderate recommendation).
  3.4. Behavior Modification
    3.4.1. Behavioral modifications, including regular and adequate sleep, reduction of screen time, and managing psychosocial stressors, should be tailored to the individual child and their family’s needs. Counseling or psychological support may be warranted (level D; weak recommendation).
  3.5. Medications
    3.5.1. We do not recommend the use of anti-obesity medications in children with CKD2-5D or with a kidney transplant and O&MS (ungraded).
  3.6. Bariatric surgery
    3.6.1. Weight loss surgery may be considered in a selected subgroup of children with CKD2-5D or with a kidney transplant and O&MS when all other interventions have failed. Patients who may be considered for weight loss surgery include:
      - Adolescents with extreme obesity (BMI ≥ 40 kg/m^2^) and other comorbidities associated with long-term risks (Level C; weak recommendation)
      - Adolescents with BMI ≥ 35 kg/m^2^ with specific obesity-related comorbidities including T2DM, severe steatohepatitis, pseudotumor cerebri, and moderate-to-severe obstructive sleep apnea (Level C; weak recommendation)

### Evidence and rationale

#### Diet

In the general pediatric population, a meta-analysis on the health effects of adherence to a Mediterranean diet found improvements in BMI and waist circumference in most, but not all studies [59]. One systematic review found that the Dietary Approaches to Stop Hypertension (DASH) diet in adolescents had beneficial effects on BP, overweight and obesity [60]. A systematic review and meta-analysis on the effects of low glycemic index/low glycemic load dietary regimens provided evidence of a beneficial effect of such a diet pattern when looking for alternative regimens in the management of obese children and adolescents [61]. Whereas very low energy diets providing less than 800 kcal/day may be effective for treating children and adolescents with obesity, conclusions with respect to their overall safety are unclear [62]. While the quality of evidence is generally low, findings of one meta-analysis involving over 400 children and adolescents from 9 randomized controlled trials (RCTs) suggested that modulation of gut microbiota through probiotics and synbiotic supplements did not have favorable effects in the management of overweight and obese children and adolescents [63].

No single dietary modification is effective in the management of O&MS in all children with CKD. Data from the CKiD cohort suggests that several aspects of the usual dietary intake of these children put them at risk for O&MS. On average, they were found to have intakes in excess of age-appropriate amounts for energy, sodium, and phosphorus [42, 64]. Fast foods contributed a large percentage of this intake. Food sources with the highest amounts of dietary fats, primarily saturated fat, were processed foods. Consumption of excess “empty calories” foods was also prevalent [42]. There is no evidence supporting the use of a specific diet pattern in children with CKD2-5D or with a kidney transplant and O&MS. In a single-center study, yearly tailored dietary assessment and counselling had a poor effect on preventing post-transplantation weight gain in children, suggesting the need for more comprehensive interventions to reduce post-transplant obesity [65]. Healthy dietary patterns, including Mediterranean and DASH diets, have been associated with reduced chronic disease incidence and improved CV and kidney outcomes in the general adult population [66, 67]. In a recent meta-analysis, higher adherence to a healthy dietary pattern was associated with a 28% lower risk of kidney disease in an analysis of 8 prospective adult cohort studies [68].

Cardio-protective mechanisms of the Mediterranean and DASH diets are largely driven by improved lipid, glycemic, and BP control. In adults, the DASH, Nordic, and Mediterranean diets have been shown to significantly lower systolic BP and diastolic BP [69]. A Cochrane review on dietary interventions in adults with CKD, including a carbohydrate-restricted, low-iron, polyphenol-enriched diet; increased fruit and vegetable intake; Mediterranean diet; and high-protein, low-carbohydrate diet, showed no impact on BMI, but beneficial effects on BP and lipid levels. Of note, all the studies included in the Cochrane review were designed to assess CV and total mortality but failed to show any effect of dietary modification on primary outcome [70].

Although not specific to pediatrics, the European Renal Nutrition (ERN) Working Group of the European Renal Association-European Dialysis Transplant Association (ERA-EDTA) promotes the Mediterranean diet as the preferred diet for adults with CKD [71]. Adjustments through choice of low-potassium fruits and vegetables and potassium-lowering medications may be necessary. The intake of a more favorable fat profile, low glycemic index, high fruit, vegetable and fiber intake, low-sodium intake, reduced acid load, and the promotion of non-processed foods are all features consistent with standard recommendations for obesity prevention and improved kidney health.

In healthy children with O&MS, attainment of BMI below overweight threshold is usually targeted [39]. A BMI reduction of −0.25 SDS has been reported to result in significantly improved BP, triglycerides, and HDL levels [72]. Still, individualized energy intake according to age, CKD stage, dialysis, and comorbidities in order to achieve normal growth should be provided according to PRNT recommendations for energy requirements in children with CKD2-5D [17]. In children on PD, the energy intake from dialysate must be considered, with a report of 9.08 ± 4.13 kcal/kg/day contributing to total energy intake [73], with variation depending on peritoneal glucose exposure and peritoneal membrane transporter status. Dietary modifications for CKD and enteral tube feeding to protect from underweight may also increase the risk of developing obesity if not carefully managed [4, 74]; these issues have been discussed in the PRNT’s recommendations on enteral feeding [74].

#### Physical activity

In the general pediatric population, a meta-analysis of studies including 2239 overweight and obese children and adolescents showed that thrice weekly 50-min sessions of aerobic and combined aerobic and strength training exercise were associated with a reduction in BMI z-score [75]. There is a consistent favorable impact of exercise on both anthropometric and cardio-metabolic parameters, with variable improvements in body weight, body fat percentage, waist circumference, and cardio-metabolic parameters [76]. A systematic review found that structured physical activity interventions decreased energy intake in obese but otherwise healthy adolescents [77]. Expert recommendations for physical activity for healthy children and adolescents from numerous professional organizations suggest 60 min of physical activity daily, ideally with a combination of aerobic, stretching, flexibility, and balance, with muscle and bone strengthening activities included [19, 78].

Compared to the 2012 NHANES survey, adolescents in the CKiD study self-reported engaging in significantly less physical activity and greater screen time compared to healthy adolescents in the NHANES population [44]. Only 13% of the children with CKD met current physical activity recommendations and 98% exceeded recommended daily screen time. Screen time is associated with obesity and lower GFR. Similar results have been reported in a single-center study that included non-dialysis and dialysis patients and kidney transplant recipients using objective measurements of physical activity and performance by 7-day pedometer assessment and 6-min walk distance [79]. Cardiorespiratory fitness by treadmill exercise testing (VO_2_max) is lower in children with non-dialysis CKD3-4, on dialysis and after kidney transplantation, and is associated with the presence of obesity and cardio-metabolic risk factors [80, 81]. The exercise capacity does not seem to improve after kidney transplantation and negatively associates with increase in fat weight [82, 83].

Kidney function is a key determinant of exercise capacity in CKD patients. Low physical activity in children with CKD may be explained by poor exercise tolerance and early fatigue due to anemia, inflammation, and the effects of uremia and metabolic acidosis on skeletal muscles and the heart [84, 85]. Advanced CKD is associated with deficits in lean leg mass and muscle wasting [86]. There is evidence that exercise may result in a transient worsening of pre-existing metabolic acidosis which may, in turn, reverse the normal anabolic effects of exercise [85].

Understanding an individual’s physical capability is paramount when advising an exercise prescription which, ideally, should involve the child and the family and be customized to their individual capacity and comorbidities. Aerobic physical activity may be advised daily. Also, muscle strengthening activities 2–3 times per week, or as tolerated, should be considered to improve muscle mass and enhance individual tolerance [87]. Small studies in children with CKD have shown mixed results with regards to feasibility of exercise programs and long-term adherence [88, 89]. Although evidence is limited, intradialytic exercise may be considered as an option for children on maintenance hemodialysis. Ongoing and consistent attention to adequate physical activity must be addressed and re-enforced during clinic visits.

#### Behavior modification

Studies in the general pediatric population suggest that longer sleep duration is associated with lower adiposity scores and better quality of life [90]. An overview of 39 systematic reviews reported a strong association between sleep duration and reduced adiposity and improved emotional outcomes, but the effect on cardio-metabolic outcomes was variable [91]. In addition to sleep duration, there are reports of sleep quality and its association with obesity [87]. Sleep disturbances are common in children with CKD [92], but there are no studies on the effect of poor sleep duration or sleep quality on the incidence of O&MS in this patient group.

Systematic reviews in the general pediatric population indicate an association between increased screen time and poorer diet [93], with increased energy intake and increased adiposity [94], but only limited evidence showing a direct relationship with cardio-metabolic outcomes [95, 96]. In line with this and as noted previously, data from the CKiD cohort showed that increased screen time is associated with increased rates of obesity [44].

Psychosocial stressors may lead to emotional or comfort eating, lack of sleep, impulsive and selective food behaviors, as well as higher levels of cortisol and catecholamine secretion, contributing to the development of central O&MS [97]. Although there are no studies of the impact of psychological stress and emotional response on O&MS in children with CKD, there are several such studies involving adult CKD patients. While there is awareness of weight problems and the benefit of weight loss, adult dialysis patients report barriers to successfully addressing weight related issues such as lack of motivation, time, money, resources, and knowledge regarding strategies to achieve weight loss goals [98].

#### Medications

Currently, five anti-obesity drugs have been approved by the US Food and Drug Administration (FDA) for long-term use in adults without kidney disorders. Randomized trials in adults suggest that the weight lowering potential of these five anti-obesity drugs appears to be in the following descending order: phentermine/topiramate, liraglutide, naltrexone/bupropion, lorcaserin, and orlistat [99]. Lorcaserin and orlistat allow easier dosing and better tolerability compared to other approved anti-obesity drugs.

There are no RCTs of anti-obesity medications in children or adults with kidney disorders. In obese adolescents without kidney disorders, RCTs have shown conflicting results. Although orlistat did not significantly reduce BMI in comparison with placebo at 6 months [100], a longer-term RCT found that in combination with diet, exercise, and behavioral modification, orlistat did significantly improve weight management compared with placebo at 1-year follow-up [101]. Importantly, orlistat has been implicated in causing acute kidney injury in patients with CKD with calcium oxalate crystal deposition in the lumen of the kidney tubules. Given these safety issues and the absence of studies in this cohort, we do not recommend the use of orlistat or other anti-obesity medications in children with kidney disorders.

#### Bariatric surgery

There is emerging evidence that bariatric surgery (BS) in obese adolescents with and without diabetes may prevent or even reverse kidney abnormalities. The results from the Teen Longitudinal Assessment of BS (Teen-LABS) study, a multicenter study of 242 adolescents who underwent bariatric surgery, showed improvement in albuminuria and eGFR. In another study, 5-year kidney outcomes were compared in adolescents with severe obesity and T2DM enrolled in the Teen-LABS and the Treatment Options for T2DM in Adolescents and Youth (TODAY) studies [102]. At baseline, elevated albuminuria was present in 21% and it increased to 43% at 5 years in TODAY participants. In contrast, albuminuria decreased from 27% of Teen-LABS participants prior to surgery to 5% at 5 years’ follow-up. At 5 years, there were 27-fold higher odds of diabetic kidney disease (DKD) in TODAY participants compared to Teen-LABS participants in adjusted analyses. A recent comparison of BS in adults (LABS study) and adolescents (Teen-LABS study) showed that adolescents experienced earlier remission of elevated albuminuria than adults [103]. These data indicate that BS improves kidney outcomes in youths with severe obesity, including those with T2DM.

The benefits of BS have also been assessed in adults with CKD, but there is no data in children. Available data from retrospective studies in adults with CKD conclude that CKD or chronic dialysis should not be a contraindication for BS [104, 105] and that BS may improve transplant candidacy [106].

4. How are MS components managed?
  4.1. Hypertension
    4.1.1. We suggest avoiding excessive sodium intake in all children with CKD2-5D or with a kidney transplant and O&MS to prevent hypertension and to further reduce dietary sodium intake in those with hypertension (level B; moderate recommendation).
  4.2. Dyslipidemia
    4.2.1. We suggest dietary interventions and lifestyle modifications to treat dyslipidemia in children with CKD2-5D or with a kidney transplant and O&MS (level D; weak recommendation).
    4.2.2. We do not suggest the routine use of statins and other lipid lowering agents (level D; weak recommendation).
  4.3. Diabetes/glucose intolerance
    4.3.1. We suggest that all children with CKD2-5D or with a kidney transplant and O&MS receive comprehensive education to manage abnormal glucose metabolism (level D; weak recommendation).
    4.3.2. Medications that are known to cause abnormal glucose must be reviewed and the dose adjusted, if appropriate (ungraded).

### Evidence and rationale

#### Hypertension

Trials investigating the role of dietary sodium intake in children with kidney diseases and O&MS are lacking. However, a single interventional trial [107] and several cross-sectional studies [108] in children without kidney disease showed that BP in obese children is characterized by a higher sodium sensitivity than in non-obese children. In the general pediatric population, a significant association between sodium intake and BP has been reported by multiple observational and intervention studies [109]. Based on the available evidence and in accordance with KDIGO and Kidney Disease Outcomes Quality Initiative (KDOQI) guidelines, we suggest not to exceed age-based recommended daily sodium intake for healthy children [110] in all patients with O&MS to prevent hypertension and to further reduce dietary sodium intake in case of high BP. An RCT on the use of the DASH diet in adolescents with newly diagnosed hypertension supports a beneficial long-term effect on systolic BP over nutrition counseling according to the National High BP Education Program [111]. The pharmacological approach to hypertension in children with O&MS and kidney diseases is beyond the scope of this CPR.

#### Dyslipidemia

Nutrition and exercise are the first-line treatments for the management of dyslipidemia in children with CKD and O&MS, as in healthy children. There are no RCTs evaluating the use of statins in children with O&MS with or without kidney disease. Two small RCTs have compared statins to placebo in children with various kidney disorders (mainly nephrotic syndrome), with conflicting results in terms of lipid profile improvement [112, 113]. No long-term studies looking at hard outcomes are available. Various systematic reviews have assessed statin therapy in adult patients with CKD or following kidney transplantation. Only in patients with CKD not on dialysis and without CV disease at baseline was statin therapy found to reduce death, major CV events, and myocardial infarction [114]. In our view, it is hard to extrapolate these adult studies to pediatric patients.

There is a lack of consensus among current guidelines. The 2011 Expert Panel on integrated guidelines for CV health risk reduction in children and adolescents of the National Heart, Lung, and Blood Institute recommended the initiation of statins in patients between 10 and 21 years of age with persistently elevated low-density lipoprotein (LDL) cholesterol after 6 months of dietary management [19]. The 2013 KDIGO clinical practice guideline for lipid management in CKD did not suggest the initiation of statins or a statin/ezetimibe combination in CKD patients under 18 years due to the lack of long-term safety data and the low level of evidence for benefit [115]. Another debated issue is the pharmacological treatment of hypertriglyceridemia in children with CKD, in particular with fibric acid derivatives, niacin, and fish oil [22]. Once again, the evidence regarding the use of these agents is extremely scarce and not specific for children with O&MS and CKD.

#### Diabetes/glucose intolerance

Weight management through lifestyle modification, including nutrition and exercise, is the cornerstone of treatment for O&MS and improvement of glucose metabolism in children with CKD. Children with risk factors for new onset diabetes after transplantation (NODAT) and post-transplant glucose intolerance, including African-American race, obesity, family history of diabetes, and those on immunosuppressant regimens with steroids, calcineurin inhibitors, and sirolimus, would especially benefit from early implementation of lifestyle modification [47]. All CKD patients with abnormal glucose metabolism should receive comprehensive self-management education. Examples of an education program might include, but not be limited to, information about T2DM and how to prevent it: healthy eating habits, portion sizes, and reading food labels. The program should be individualized according to the child and the family’s abilities and preferences. Health care providers should assess potential barriers including food insecurity, home stability, and financial difficulties. Psychological assessment for symptoms of depression and eating disorders should be performed. Patients with pre-diabetes who fail lifestyle modification, or those diagnosed with diabetes, should be referred to an endocrinologist for potential pharmacological treatment of abnormal glucose metabolism. Decreasing the dose, stopping, or changing medications associated with abnormal glucose metabolism (e.g., steroids, calcineurin inhibitors) should be considered, if possible [47].

5. How can O&MS be prevented?
  5.1. We recommend a healthy diet, regular physical activity, and other behavioral modifications to prevent O&MS (level D; weak recommendation).

#### Evidence and rationale

Prevention of pediatric obesity through promotion of a healthy diet, activity, and healthy lifestyle is paramount (Fig. 1). The principles outlined in the management of O&MS are equally applicable to prevention. This is of particular importance in children with nephrotic syndrome or after kidney transplantation on steroid regimens. Dietary principles should follow the recommendations in statement 3.2. No single diet plan works best for all patients, but concepts such as avoiding excess energy intake by limiting sugar and total fat intake (favoring unsaturated fats and, in particular, higher omega-3 fats over saturated fats), promoting fiber, fruits, vegetables, pulses, and whole grains, while considering the individual’s age and CKD-appropriate requirements, seem appropriate for prevention of O&MS in children with CKD. Physical activity may help prevent O&MS and is important for overall health. Recommendations, previously discussed in statement 3.3 related to physical activity, should be followed adjusting as needed for age, CKD stage, and comorbidities. Limiting screen time and other positive psychosocial influences, such as family meals, mindfulness when eating, stress, and emotional management, are prudent to prevent the onset of O&MS. Awareness and early recognition of evolving MS components may reinforce implementation of lifestyle modifications and guide clinical decision-making.

#### Results of the Delphi survey

There were 50 responses to the electronic Delphi survey with joint responses submitted by some dietitians and physicians from the same facility. All professionals who completed the survey are listed in “Participants in the Delphi survey.”

The 22 clinical practice recommendation statements received an overall 87% consensus with a “strongly agree” or “agree” response and 11% with a “neutral” response; these largely reflected the wide variations in practice in the absence of robust evidence. Two statements did not reach the stipulated 70% level of consensus. The Taskforce members reviewed the comments and agreed that the statements did not require changing as the GRADE clearly reflects the low level of evidence, indicating that these statements are based on expert opinion.

The highest “disagree and strongly disagree” rate was in response to statement 3.6.1 on bariatric surgery. On careful review of the literature and discussion within the Taskforce team, it was agreed that despite country policy, legal stipulations and few centers with the relevant experience, bariatric surgery may be considered in a selected subgroup of children with CKD2-5D or with a kidney transplant and O&MS when all other interventions have failed. Based on suggestions from Delphi respondents, minor rewording of statements and further clarification to the text were done.

### Summary of recommendations

A summary of recommendations is provided in Table 4.

**Table 4:** Summary of recommendations

		Recommendations	Grade
1.	How is O&MS defined?	1.1 Children aged 2-5 years:	
		1.1.1 We define overweight as weight-for-height for age > +2SD, using the World Health Organization (WHO) child growth standard chart.	
		1.1.2 We define obesity as weight-for-height for age > +3SD, using the WHO child growth standard chart. 1.2 Children aged > 5 years:	
		1.2.1 We define overweight as body mass index (BMI) for age > +1SD, equivalent to BMI > 25 kg/m^2^ at 19 years, using the WHO growth reference chart or a country-specific growth chart.	
		1.2.2 We define obesity as BMI for age > +2SD, equivalent to BMI > 30 kg/m^2^ at 19 years, using the WHO growth reference chart or a country-specific growth chart.	
		1.3 Children aged 2-18 years:	
		1.3.1 We define metabolic syndrome as the presence of overweight or obesity and at least 2 of 4 additional CV risk factors: a .Systolic and/or diastolic office blood pressure (BP) ≥ 90^th^ centile for age, sex and height or ≥ 130/80mmHg, whichever is lower, or on anti-hypertensive medication b. Fasting triglycerides ≥ 100 mg/dL (1.1 mmol/L) if age < 10 years, or ≥ 130 mg/dL (1.5 mmol/L) if age ≥ 10 years c. Fasting high-density lipoprotein (HDL) < 40 mg/dL (1.03 mmol/L) d. Fasting serum glucose ≥ 100 mg/dL (5.6 mmol/L) or known type 2 diabetes mellitus (T2DM)	
		1.3.2 We recommend using BMI-height-age to define overweight or obesity in children who are below the 3^rd^ centile for height and have not reached their final adult height	Level B; moderate recommendation
2.	How is O&MS assessed?	2.1 Calculate BMI or weight-for-height and plot on centile growth charts.	Level A; strong recommendation
		2.1.1 Calculate z-scores [standard deviation scores (SDS)] to complement growth chart plots.	Level X; strong recommendation
		2.1.2 Utilize trends in growth parameters to assist clinical decision-making.	Level D; weak recommendation
		2.2 Measure BP, fasting TG, HDL and glucose levels in children with CKD2-5D and after transplantation if BMI > +1 SD.	Level A; strong recommendation
		2.3 Evaluate for MS risk factors, including focused history and physical exam, biochemical measurements for comorbidities and assessment of cardio-metabolic risk factors.	Level C; weak recommendation
		2.4 Evaluate lifestyle habits, including diet, physical activity, sleep and screen time.	Level C; weak recommendation
		2.5 The frequency of assessment should be individualized based on the child’s CV risk factors, disease severity and progression and the presence of comorbidities	Ungraded
3.	How is O&MS managed?	3.1 We suggest a comprehensive multicomponent intervention that includes a nutrition care plan, physical activity prescription and behavioral modification to reduce BMI and improve components of the MS.	Ungraded
		3.2 Diet	Ungraded
		3.2.1 We recommend an individualized energy intake, adjusted for age, CKD stage, dialysis and comorbidities, to achieve weight loss or weight maintenance in children without compromising their nutrition.	
		3.2.2 The nutrition care plan should aim to improve the overall diet quality, with an emphasis on an intake comprised primarily of fruits and vegetables, whole grains, low- or non-fat dairy products, pulses (peas, beans, lentils), fish and lean meat, and avoidance of sugar-sweetened beverages, highly processed foods and foods high in saturated fat.	Level B; weak recommendation
		3.2.3 In children who are enterally tube fed, the energy content of the formula must be frequently reviewed and adjusted to avoid development of underweight or overweight.	Level B; moderate recommendation
		3.3 Physical Activity	
		3.3.1 We recommend that children engage in daily physical activity with intensity and duration individualized according to age, physical tolerance, CKD stage, and comorbidities.	Level B; moderate recommendation
		3.4 Behavior Modification	
		3.4.1 Behavioral modifications, including regular and adequate sleep, reduction of screen time and managing psychosocial stressors, should be tailored to the individual child and their family’s needs. Counselling or psychological support may be warranted.	Level D; weak recommendation
		3.5 Medications	
		3.5.1 We do not recommend the use of anti-obesity medications in children with CKD2-5D or with a kidney transplant and O&MS.	Ungraded
		3.6 Bariatric Surgery	
		3.6.1 Weight loss surgery may be considered in a selected subgroup of children with CKD2-5D or with a kidney transplant and O&MS when all other interventions have failed. Patients who may be considered for weight loss surgery include:	
		a. adolescents with extreme obesity (BMI ≥ 40 kg/m2) and other comorbidities associated with long-term risks	Level C; weak recommendation
		b. adolescents with BMI ≥ 35 kg/m2 with specific obesity-related comorbidities including T2DM, severe steatohepatitis, pseudotumor cerebri, and moderate-to-severe obstructive sleep apnea	Level C; weak recommendation
4.	How are MS components managed?	4.1 Hypertension	
		4.1.1 We suggest avoiding excessive sodium intake in all children with CKD2-5D or with a kidney transplant and O&MS to prevent hypertension, and to further reduce dietary sodium intake in those with hypertension.	Level B; moderate recommendation
		4.2 Dyslipidemia	
		4.2.1 We suggest dietary interventions and lifestyle modifications to treat dyslipidemia in children with CKD2-5D or with a kidney transplant and O&MS.	Level D; weak recommendation
		4.2.2 We do not suggest the routine use of statins and other lipid lowering agents.	Level D; weak recommendation
		4.3 Diabetes/glucose intolerance	
		4.3.1 We suggest that all children with CKD2-5D or with a kidney transplant and O&MS receive comprehensive education to manage abnormal glucose metabolism.	Level D; weak recommendation
		4.3.2 Medications that are known to cause abnormal glucose metabolism must be reviewed and the dose adjusted, if appropriate.	Ungraded
5.	How can O&MS be prevented?	5.1 We recommend a healthy diet, regular physical activity and other behavioral modifications to prevent O&MS.	Level D; weak recommendation

### Research recommendations

We recommend the following areas of study to provide future evidence-based recommendations for O&MS in children with CKD2-5D and after transplantation:

- To investigate the role of waist-to-height ratio to assess central adiposity, both its practical application and its ability to provide additional guidance regarding management and outcomes compared to BMI.
- To investigate the utility of different measurements of adiposity, BMI, and weight-for-length (WFL), in the assessment of overweight and obesity in infants with CKD and their associations with clinical outcomes.
- To investigate the use of handgrip strength to assess muscle deficits and obese sarcopenia and the effectiveness of interventions to correct these muscle disorders.
- To examine the possible role of adipokines including ghrelin and leptin in the pathogenesis of protein energy wasting and pathophysiology of obese sarcopenia in children with CKD.
- To increase evidence on the impact of O&MS on target organ damage other than LVH, including cIMT and PWV, as well as the effect of treatment of O&MS on short- and long-term CV outcomes.
- To investigate the role of management of uric acid levels in the treatment of O&MS.
- To conduct controlled trials on the effect of specific diet plans, notably Mediterranean and DASH diets, in large pediatric CKD populations.
- To assess the role of the microbiome on the prevalence of O&MS in children with CKD and potential of modification by different diet patterns.
- To study the effectiveness of behavioral modification interventions on O&MS prevention and treatment in pediatric CKD patients and after kidney transplantation.

## Supplementary Information

ESM 1 (DOCX 354 kb)

## Declarations

### Disclaimer

The Pediatric Renal Nutrition Taskforce wishes to confirm that Vitaflo has not influenced the development or content of these Clinical Practice Recommendations. The views expressed are those of the authors and not necessarily those of the NHS, the NIHR, or the Department of Health and Social Care.
