# Supplementary material for: Assessment and management of obesity and metabolic syndrome in children with CKD stages 2–5 on dialysis and after kidney transplantation—clinical practice recommendations from the Pediatric Renal Nutrition Taskforce
Source: Pediatr Nephrol. 2021 Aug 10;37(1):1–20. doi: 10.1007/s00467-021-05148-y (PMC8674169; doi:10.1007/s00467-021-05148-y)
Supplement: Supplementary file 1 — (DOCX 354 kb) [file 467_2021_5148_MOESM1_ESM.docx]

**Supplementary Material**

**PICO questions and literature search**

**Developing the PICO questions**

We developed PICO questions in order to develop recommendations that provide specific actionable advice, with specification of the patient group (P) to whom the statement would apply; the intervention (I) being considered; the comparator (C) (which may be “no action” or an alternative intervention); and the outcomes (O) affected by the intervention. The PICO terms were:

*Population:* Children from 2 years to 18 years of age with CKD2-5, on dialysis, and after kidney transplantation. Children with CKD1 have been excluded.

*Intervention:* Prevention or treatment of O&MS in children with CKD2-5D and after transplantation.

*Comparator:* Strategies for prevention or management of O&MS in age- and sex-matched general pediatric populations, adults with CKD who have O&MS, or no comparator.

*Outcome:* Weight reduction, regression of O&MS and associated adverse outcomes, including CV risk factors, CV target organ damage, CKD progression, access to transplantation, graft function, mortality.

Obesity in children under 2 years is only addressed briefly in the document, as there are no outcome measures for children in this age group and there is currently no accepted definition for adiposity in children under age 2 years. The International Pediatric Peritoneal Dialysis Network (IPPDN) has shown that 20% of infants receiving PD have a BMI-SDS > 95^th^ centile (> 1.645 SDS) [1, 2], with the highest prevalence in the Middle East and US, in keeping with national obesity prevalence rates. Gastrostomy feeding was the most significant risk factor for obesity. Finally, with obesity associated with increased mortality in children under 5 years on PD, careful attention to the nutritional management of those fed by enteral tube is required [3]. Obesity in children under 2 years remains an important area of research, and has been included as a Research recommendation.

**Literature search**

An evidence search was performed for papers published from 1980 through September 2020. Details of the literature search are described in Supplementary Table 1. In children with CKD, all studies pertaining to O&MS were included, with retrospective studies limited to those with more than 20 children. Given the paucity of high-quality studies in this field, the literature search was expanded to include meta-analyses in adults with obesity and/or MS and CKD2-5D and after transplantation, as well as meta-analyses and randomized controlled trials (RCTs) conducted in the general pediatric population with O&MS. The management of hypertension beyond its relation with dietary sodium is outside the scope of this document. The management of sodium and fluid in children with CKD2-5D and after transplantation will be addressed in a separate document from the PRNT.

“Given the paucity of data in children with CKD, the recommendations provided in this CPR are based on studies performed in healthy children and adolescents as well as those with CKD. Meta-analyses in adults with CKD have also been included in the setting of more limited pediatric data, but clearly indicating that these data must be extrapolated to the pediatric population with caution.”

After a critical review of the literature for each PICO question, CPRs were derived and graded using the American Academy of Pediatrics (AAP) grading matrix (Supplementary Table 2) [4]. Given the very low grade of evidence for most recommendations, we conducted a Delphi survey (e-questionnaire) to attain consensus from experts in the field, as previously described [5].

**References**

1. Rees L, Azocar M, Borzych D, Watson AR, Büscher A, Edefonti A, Bilge I, Askenazi D, Leozappa G, Gonzales C, van Hoeck K, Secker D, Zurowska A, Rönnholm K, Bouts AH, Stewart H, Ariceta G, Ranchin B, Warady BA, Schaefer F (2011) Growth in very young children undergoing chronic peritoneal dialysis. J Am Soc Nephrol 22:2303-2312

2. Schaefer F, Benner L, Borzych-Duzalka D, Zaritsky J, Xu H, Rees L, Antonio ZL, Serdaroglu E, Hooman N, Patel H, Sever L, Vondrak K, Flynn J, Rebori A, Wong W, Holtta T, Yildirim ZY, Ranchin B, Grenda R, Testa S, Drozdz D, Szabo AJ, Eid L, Basu B, Vitkevic R, Wong C, Pottoore SJ, Muller D, Dusunsel R, Celedon CG, Fila M, Sartz L, Sander A, Warady BA (2019) Global Variation of Nutritional Status in Children Undergoing Chronic Peritoneal Dialysis: A Longitudinal Study of the International Pediatric Peritoneal Dialysis Network. Sci Rep 9:4886

3. Rees L, Shaw V, Qizalbash L, Anderson C, Desloovere A, Greenbaum L, Haffner D, Nelms C, Oosterveld M, Paglialonga F, Polderman N, Renken-Terhaerdt J, Tuokkola J, Warady B, Walle JV, Shroff R (2021) Delivery of a nutritional prescription by enteral tube feeding in children with chronic kidney disease stages 2-5 and on dialysis-clinical practice recommendations from the Pediatric Renal Nutrition Taskforce. Pediatr Nephrol 36:187-204

4. American Academy of Pediatrics Steering Committee on Quality Improvement and Management (2004) Classifying recommendations for clinical practice guidelines. Pediatrics 114:874-877

5. Shaw V, Polderman N, Renken-Terhaerdt J, Paglialonga F, Oosterveld M, Tuokkola J, Anderson C, Desloovere A, Greenbaum L, Haffner D, Nelms C, Qizalbash L, Vande Walle J, Warady B, Shroff R, Rees L (2020) Energy and protein requirements for children with CKD stages 2-5 and on dialysis-clinical practice recommendations from the Pediatric Renal Nutrition Taskforce. Pediatr Nephrol 35:519-531

**Supplementary Table 1** Search term strategy used in the literature review

**Search terms**

| 1 | kidney disease | chronic kidney disease | kidney dysfunction | CKD | ESRD and ESKD | pre dialysis | renal replacement therapy | dialysis |
| --- | --- | --- | --- | --- | --- | --- | --- | --- |
|  | peritoneal dialysis | CAPD | APD | hemodialysis | hemodialysis | kidney transplantation | renal  transplantation | IPPN |
|  | CKiD Study | ESPN/ERA-EDTA Registry | NAPRTCS | USRD | ANZDATA |  |  |  |
| 2 | Overweight | Obesity | Metabolic syndrome | Body mass index | Waist circumference | Waist-to-height ratio | Hypertension | Blood pressure |
|  | Lipids | Cholesterol | Triglycerides | HDL | Glucose intolerance | Glucose | Insulin | Diabetes |
|  | Protein energy wasting | Muscle wasting | sarcopenia | Uric acid |  |  |  |  |
| 3 | Nutrition | Diet | Dietary management | Dietary restriction | Diet/food recall | Food record/diary | Mediterranean diet | DASH diet |
|  | Intermittent fasting | Ketogenic diet | Macronutrients | Dietary energy density | Meal replacements | Plant-based diet | Plant sterols | Synbiotic supplementation |
|  | Vegan diet | Vegetarian diet | Very low energy diet | Vitamin D | Prevention | Sodium | Sodium restriction | Sodium intake |
|  | Omega fats | Fiber | Sleep | Sleep duration | Lipids | Hyperlipidemia | Exercise | Physical activity |

**Search methods:**

- From 1980 through September 2020
- Electronic search using PubMed, Medline, Embase, Cochrane library.
- Reference list from review articles and clinical practice guidelines (including KDOQI & KDIGO)

**Supplementary Table 2** American Academy of Pediatrics grading matrix


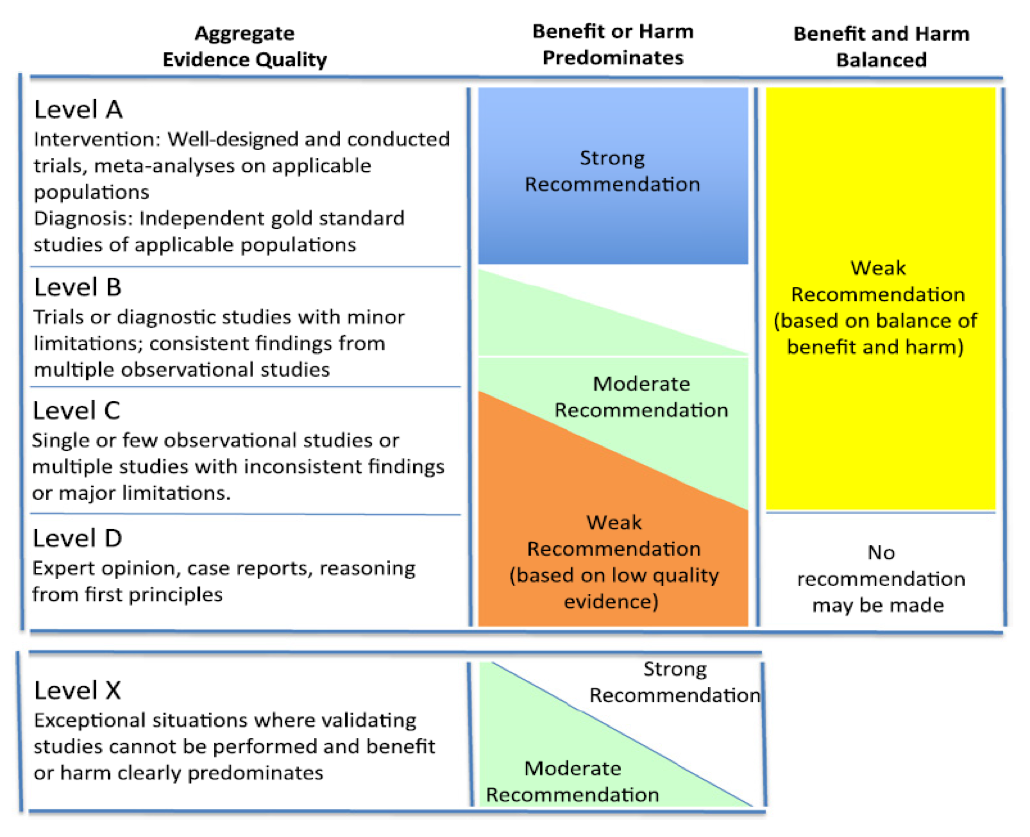


**Supplementary Table 3** Agreement and disagreement responses to the Delphi survey

| **Recommendations** | **Agree/Disagree *** |
| --- | --- |
| - 1. We recommend using BMI-height-age to define overweight or obesity in children who are below the 3^rd^ centile for height and have not reached their final adult height | 83%/3% |
| 2.1. Calculate BMI or weight-for-height and plot on centile growth charts. | 93%/0% |
| 2.1.1. Calculate z-scores [standard deviation scores (SDS)] to complement growth chart plots. | 90%/3% |
| 2.1.2. Utilize trends in growth parameters to assist clinical decision-making. | 86%/0% |
| 2.2. Measure BP, fasting TG, HDL and glucose levels in children with CKD2-5D and after transplantation if BMI > +1 SD. | 93%/0% |
| 2.3. Evaluate for MS risk factors, including focused history and physical exam, biochemical measurements for comorbidities and assessment of cardio-metabolic risk factors. | 90% /3% |
| 2.4. Evaluate lifestyle habits, including diet, physical activity, sleep and screen time. | 93%/0% |
| 2.5. The frequency of assessment should be individualized based on the child’s CV risk factors, disease severity and progression and the presence of comorbidities | 86%/3% |
| 3.1. We suggest a comprehensive multicomponent intervention that includes a nutrition care plan, physical activity prescription and behavioral modification to reduce BMI and improve components of the MS. | 97%/0% |
| 3.2.1. We recommend an individualized energy intake, adjusted for age, CKD stage, dialysis and comorbidities, to achieve weight loss or weight maintenance in children without compromising their nutrition. | 97%/0% |
| 3.2.2. The nutrition care plan should aim to improve the overall diet quality, with an emphasis on an intake comprised primarily of fruits and vegetables, whole grains, low- or non-fat dairy products, pulses (peas, beans, lentils), fish and lean meat, and avoidance of sugar-sweetened beverages, highly processed foods and foods high in saturated fat. | 93%/3% |
| 3.2.3. In children who are enterally tube fed, the energy content of the formula must be frequently reviewed and adjusted to avoid development of underweight or overweight. | 100%/0% |
| 3.3.1.   We recommend that children engage in daily physical activity with intensity and duration individualized according to age, physical tolerance, CKD stage, and comorbidities. | 97%/0% |
| 3.4.1. Behavioral modifications, including regular and adequate sleep, reduction of screen time and managing psychosocial stressors, should be tailored to the individual child and their family’s needs. Counselling or psychological support may be warranted. | 83%/3% |
| 3.5.1. We do not recommend the use of anti-obesity medications in children with CKD2-5D or with a kidney transplant and O&MS. | 72%/3% |
| 3.6.1. Weight loss surgery may be considered in a selected subgroup of children with CKD2-5D or with a kidney transplant and O&MS when all other interventions have failed. Patients who may be considered for weight loss surgery include:  a. adolescents with extreme obesity (BMI ≥ 40 kg/m2) and other comorbidities associated with long‐term risks  b. adolescents with BMI ≥ 35 kg/m2 with specific obesity‐related comorbidities including T2DM, severe steatohepatitis, pseudotumor cerebri, and moderate‐to‐severe obstructive sleep apnea | 41%/17% |
| 4.1.1. We suggest avoiding excessive sodium intake in all children with CKD2-5D or with a kidney transplant and O&MS to prevent hypertension, and to further reduce dietary sodium intake in those with hypertension. | 86%/3% |
| 4.2.1. We suggest dietary interventions and lifestyle modifications to treat dyslipidemia in children with CKD2-5D or with a kidney transplant and O&MS. | 86%/0% |
| 4.2.2. We do not suggest the routine use of statins and other lipid lowering agents. | 69%/0% |
| 4.3.1. We suggest that all children with CKD2-5D or with a kidney transplant and O&MS receive comprehensive education to manage abnormal glucose metabolism. | 93%/3% |
| 4.3.2. Medications that are known to cause abnormal glucose metabolism must be reviewed and the dose adjusted, if appropriate. | 86%/0% |
| 5.1. We recommend a healthy diet, regular physical activity and other behavioral modifications to prevent O&MS. | 100%/0% |

*The remainder of the responses were neutral.
